# An Improved YOLOv5 Model for Lithographic Hotspot Detection

**DOI:** 10.3390/mi16050568

**Published:** 2025-05-09

**Authors:** Mu Lin, Wenjing He, Jiale Liu, Fencheng Li, Jun Luo, Yijiang Shen

**Affiliations:** 1 School of Automation, Guangdong University of Technology, Mega Education Center South, Guangzhou 510006, China; 2112304195@mail2.gdut.edu.cn (W.H.); 2112304033@mail2.gdut.edu.cn (J.L.); lfc1362022@163.com (F.L.); 19974652127@163.com (J.L.); 2 Key Laboratory of Photoelectronic Imaging Technology and System of Ministry of Education of China, School of Optics and Photonics, Beijing Institute of Technology, Beijing 100081, China; 3120235097@bit.edu.cn (M.L.)

**Keywords:** lithographic hotspot detection, YOLOv5, spatial attention mechanism

## Abstract

The gap between the ever-shrinking feature size of integrated circuits and lithographic manufacturing ability is causing unwanted shape deformations of printed layout patterns. The deformation region with problematic imaging, known as a hotspot (HS), should be detected and corrected before mask manufacturing. In this paper, we propose a hotspot detection method to improve the precision and recall rate of the fatal pinching and bridging error due to the poor printability of certain layout patterns by embedding a spatial attention mechanism into the YOLOv5 model. Additionally, transfer learning and pre-trained techniques are used to expedite training convergence. Simulation results outperform the depth-based or representative machine learning-based methods on the ICCAD 2012 dataset with an average recall rate of 1, a precision rate of 0.8277 and an F1-score of 0.9057.

## 1. Introduction

Low $k_{1}$ lithography presents significant printablility challenges for 22 nm technology and beyond, with the down-scaling of feature size in chip design being reduced to a point where the design for manufacturing (DFM) is no longer optional. Diffraction of light is partially accountable for the cause of defects during the lithographic process, leading to serious image deformation being present on the substrate and further landing of defects occurring in the final elemental structure. Although various resolution enhancement techniques (RETs), such as optical proximity correction (OPC) [1] and sub-resolution assist features (SRAFs) [2], are used to improve the imaging fidelity, some layout regions may still be susceptible to the lithography process with pinching and bridging types of hotspots (HSs) [3], which is likely to produce open or short circuits. A hotspot can easily lead to functional failure and yield loss of IC chips. Therefore, in order to ensure the final performance and yield of IC chips, the detection of lithography hotspots must be carried out before the actual manufacture of ICs.

One way to detect HSs is to run lithographic simulations on a layout. The full lithography simulation [4,5] provides the most accurate detection results, but at the expense of extremely high computational complexity and a long runtime for full chip function. Alternatively, techniques such as pattern matching [6,7,8] and machine learning [9,10,11,12] have been suggested. Pattern matching is fast and accurate by comparing the image to be tested with the pre-characterized HS patterns in a library. However, it lacks the flexibility to recognize previously unseen defects. Machine learning, while being good at detecting unknown HSs with the advantage of fast detection, requires a manual or ad hoc extraction of HS feature demanding esoteric knowledge of layout properties. Hence, it is susceptible to detection omissions and false alarms [13]. Recently, deep learning [14,15,16,17] has emerged as a powerful tool to tackle the challenge of lithography HS detection. Inheriting the merits of machine learning, deep learning avoids the ambiguity associated with feature extraction. Yu and his coworkers studied the impact of convolutional neural network (CNN) hyperparameters and applied HS upsampling to address data imbalances [17]. Borisov and Scheible further investigated different data augmentation techniques to improve the classification rates for minor classes in the 2012 International Conference on Computer-Aided Design (ICCAD 2012) dataset [18]. Sim et al. transformed HSs to non-hotspots (NHSs) and vice versa via CycleGan, correcting HSs into coldspots on a synthesized dataset [19].

In general, a neural network should learn to address the complexities of underlying patterns for HS detection with regard to feature similarity and data imbalance. Lithographic printing on the wafer is susceptible to process variations where sensitive layout patterns can produce undesirable HS and NHS similarities; as a consequence, the network usually goes deep to increase the learning capacity with a greater risk of overfitting. Additionally, the data of lithography HS detection problems are highly imbalanced. As a result, most deep learning classifiers are biased with poor classification rates for minor classes. To address these issues, an improved YOLOv5 model is developed for the rapid detection of lithography HSs. By augmenting the training dataset with elaborately designed flipping, we mitigate the problem of overfitting caused by a dataset imbalance. Meanwhile, an attention mechanism is embedded in the backbone of the single-network YOLOv5 model, enabling the precise prediction and location of HSs with significantly improved sensitivity of the model to the mask pattern region. During the training process, transfer learning and a pre-trained model are used to accelerate the convergence of the HS detection network.

The rest of this paper is organized as follows: Section 2 delineates the image flipping strategy to address HS imbalances. Section 3 elaborates on the YOLOv5-based detection model with attention mechanism. Section 4 lists the experimental results of the ICCAD 2012 dataset, and Section 5 outlines the conclusions.

## 2. Data Augmentation

NHSs usually outnumber HSs by a large margin, and the presence of data imbalances can significantly impact learning within the network. From the overview of the ICCAD 2012 [20] benchmarks for 32 and 28 nm, as shown in Table 1, a higher count of NHSs compared to HSs in the training dataset is evident. For instance, in Benchmark5, there are twenty-seven times more NHSs than HSs. The imbalance within the dataset causes models to focus on the learning of NHSs, subsequently compromising the generalization ability of the detection. Consequently, data augmentation techniques are often applied to address the issue of data imbalances. It should be noted that Benchmarks 2–5 in the ICCAD 2012 dataset contain 28 nm clips, while Benchmark1 contains 32 nm clips; hence, we also combine Benchmarks 2–5 of the 28 nm clips into Benchmark6 for a further validation of HS detection.

While minority upsampling and majority downsampling [21] have shown their merits with prior data imbalance problems, their application with HS detection suffers from light diffraction in lithographic imaging and the nature of CNNs. Firstly, minority upsampling, by directly duplicating HS patterns, may lead to a unitary gradient because of excessive sample identicity straying away from the optimal solution; secondly, the number of HSs, via duplication, is still nowhere near bridging the gap with that of the NHSs. Majority or NHS downsampling, on the other hand, results in insufficient data for both NHSs and HSs, leading to CNN overfitting and degenerated performance.

**Figure 1:**
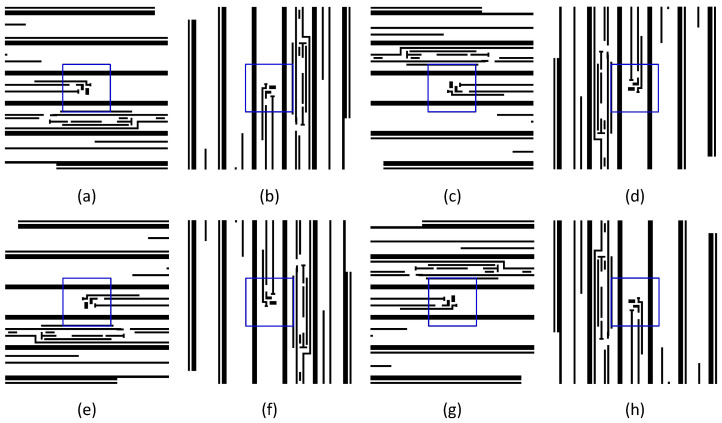
Data augmentation by flipping. (**a**) The original image. (**b**–**d**) Respective counterclockwise rotation of (**a**) by 90°, 180°, and 270°. (**e**) Horizontal flipping of (**a**). (**f**–**h**) Respective counterclockwise rotations of (**e**) by 90°, 180°, and 270°.

Assuming both horizontal and vertical symmetry in the illumination pupil, such as conventional, annular, quasar or C-quad, flipping the mask layout vertically or horizontally does not change the lithographic imaging for 32 and 28 nm lithography processes using DUV immersion technology, thus keeping the HS and NHS labeling intact. Bearing that in mind, we propose to perform data augmentation for HS sample expansion with carefully designed flipping strategies, as described in Figure 1.

As shown in Figure 1, the blue box represents the region where the lithographic hotspots are located. Figure 1a is the original image, and Figure 1b–d rotate Figure 1a counterclockwise by 90°, 180°, and 270°, respectively. Figure 1e flips Figure 1a horizontally, and Figure 1f–h are respective counterclockwise rotations of Figure 1e by 90°, 180°, and 270°. In brief, Figure 1g flips Figure 1a vertically, and Figure 1c flips Figure 1a both vertically and horizontally and data are augmented in 7 different flippings of the original image.

## 3. Detection Model for Lithographical HSs

### 3.1. YOLOv5

The YOLO series algorithm’s detection network employs a single network to determine the candidate region for HSs and identify the location and category of targets within the said region [22], demonstrating superior performance in terms of the recall rate and precision. Depending on network depths and widths, four versions of YOLOv5 are presented: YOLOv5s, YOLOv5m, YOLOv5l and YOLOv5x [23]. Generally, deeper network structures tend to have higher performance and learn more complex features, improving detection capabilities when dealing with large-scale and intricate datasets. However, deeper networks require more sophisticated optimization algorithms while also posing risks related to hidden training issues and degradation problems. Taking into account both the requirements of lithography HS detection tasks and model network depth considerations, this paper selects YOLOv5s as the model for lithography HS detection purposes.

**Figure 2:**
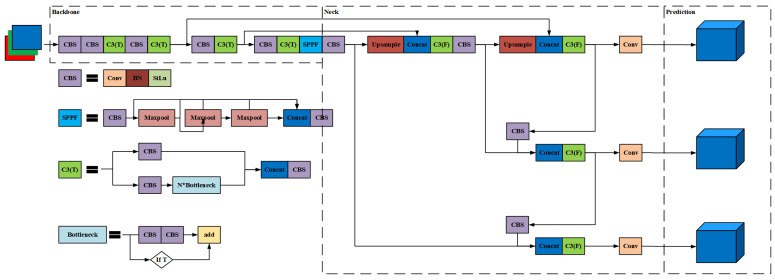
Structure of YOLOv5s.

YOLOv5s consists of Input, Backbone, Neck and Prediction components, the structure of which is schematically illustrated in Figure 2. The Input loads benchmark datasets before performing data prepossessing. The Backbone comprises the CBL, C3, and SPPF modules and is responsible for feature extraction and screening: CBL extracts features; C3 enhances network depth and receptive field to improve feature extraction capabilities; and SPPF uses max pooling to concatenate feature maps of different receptive fields for multi-scale feature fusion. The Neck concatenates different feature maps of the Backbone before passing them on to the Prediction component. Finally, HS and NHS areas are detected on the feature map, with the CIoU function [24] being used to evaluate the error between the predicted value and the actual value in the Prediction component.

### 3.2. Spatial Attention

Spatial attention (SA), being one of the attention mechanisms, provides supplementary information to the network, enabling selective focus on crucial details while disregarding secondary or irrelevant ones. In the task of lithography HS detection, due to the high similarity between the geometric features of HSs and NHSs, the network is prone to misjudging the NHSs as HSs. The introduction of SA into HS detection will enhance the feature expression of potential HS regions, thereby improving interpretability to understand and represent geometrically similar HSs and NHSs with different levels of attention.

**Figure 3:**
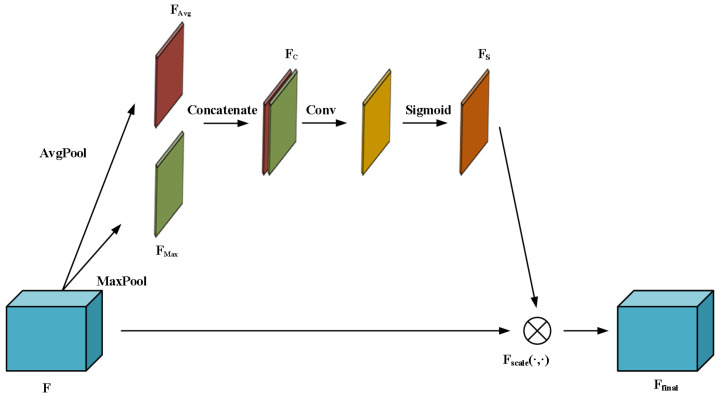
The schematic structure of the hybrid SA mechanism.

**Figure 4:**
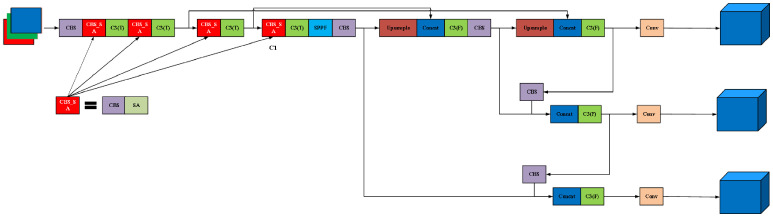
Structure of improved YOLOv5.

While average pooling is generally used for spatial information, we opt to embed the hybrid SA mechanism by exploiting the merits of max and average pooling [25] into the CBS module, where different statistical spatial distributions are offered by both pooling types. From Figure 3, which shows the schematic structure of the hybrid SA mechanism, the channel of the original feature map *F* is downsampled by average and max pooling to derive two feature maps, $F_{Avg}$ and $F_{Max}$, which are further merged into a concatenated feature map $F_{C}$ with two channels. The spatial context is captured to generate the one-channel feature map representing the weight of spatial attention by convolving $F_{C}$ with a convolution kernel. The spatial attention weights of the feature map are limited between $\left[0,1\right]$ by a sigmoid function to output $F_{s}$. Different levels of spatial importance are assigned to emphasize potential HS regions while overlooking NHS ones by the element-wise multiplication of $F_{s}$ and the original feature map *F*. The feature map $F_{final}$ with SA is as follows:

(1) $$\begin{array}{ccc} F_{final} & = & F_{scale}\left(F_{S},F\right)\\ \; & = & F_{S}⊗F\\ \; & = & F_{S}⊗\sigma \left(f^{7\times 7}\left(F_{C}\right)\right)\\ \; & = & F_{S}⊗\sigma \left(f^{7\times 7}\left([F_{Avg};F_{Max}]\right)\right), \end{array}$$

where $F_{scale}\left(\cdot ,\cdot \right)$ is the element-wise multiplication, σ(·) is the sigmoid activation, $f^{7\times 7}\left(\cdot \right)$ denotes the convolution operation with a 7 × 7 convolution kernel, and [·;·] denotes concatenation operation. We demonstrate the hybrid SA structure included in the CBS_SA module in the inset of the modified YOLOv5 model in Figure 4.

### 3.3. Transfer Learning for Data Insufficiency

While a large dataset with completely annotated data is often required for deep learning-based object detection tasks, the samples in ICCAD 2012 benchmarks even after data augmentation, as described in Section 2, are limited in size for HS detection. Hence, the training is therefore prone to overfitting. We propose to address data insufficiency by exploiting the good generalizability promised by the state-of-the-art (SOTA) models trained by the natural image dataset ImageNet [26], where model transferability is explicitly explored [27,28] and further demonstrated [29]. We fine-tune the existing SOTA model with the existing model parameters. Hence, there is no need to train the network from scratch. Thus, short training times and quick convergence are naturally expected.

We also apply pre-training to the ICCAD 2012 benchmarks by reusing the transferred knowledge of lithographic HS detection from the SOTA ImageNet model. The progressive training strategy takes advantage of both the generalizability of the SOTA ImageNet model and the geometric similarity of the 32 nm and 28 nm HS data for faster convergence and stronger robustness.

## 4. Experiment and Results

The experiment in this paper is conducted on a 6-core 3.6 GHz CPU, an NVIDIA GTX 1080Ti GPU, and using the pytorch 1.13.1 framework with CUDA version 11.7, implemented in the Python 3.8 programming language, with Windows 10 as the operating system. Benchmark statistics with data augmentation described in Section 2 are quantified in Table 2, where 90% of the benchmark samples are used for training and 10% are used for validation.

**Figure 5:**
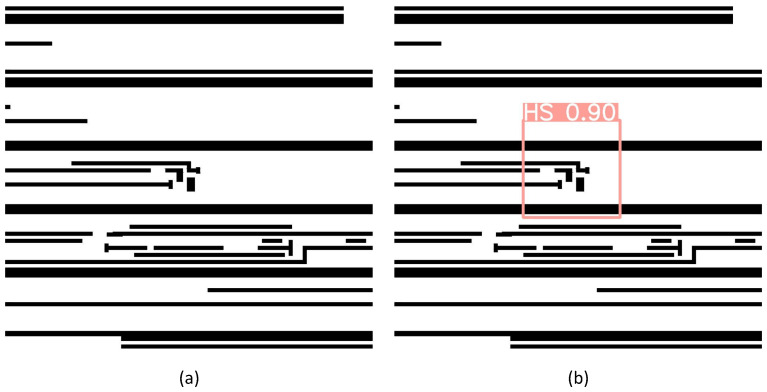
HS detection. (**a**) The candidate to be detected. (**b**) Location of and predication probability of a potential HS.

When the benchmarks containing both HS and NHS images are fed into a deep learning model for training and validation, the efficacy of the detection network is defined by its capability to accurately identify true HSs while minimizing false alarms, where excessive false alarm reports may lead to over-optimization during subsequent HS repair stages. The detection is depicted using a potential HS candidate in Figure 5a, with it being inferred with the trained model to export the location of the HS in the red square with a prediction probability of 0.9, as shown in Figure 5b.

### 4.1. Performance Indicator

We evaluate the HS detection performance with recall, precision and F1-score, which are widely used in a machine learning model performance evaluation and are defined as

(2) $$\mathrm{recall}=\frac{\mathrm{TP}}{\mathrm{TP}+\mathrm{FN}},$$

(3) $$\mathrm{precision}=\frac{\mathrm{TP}}{\mathrm{TP}+\mathrm{FP}},$$

and

(4) $$F1\text{-}\mathrm{score}=2\times \frac{\mathrm{precision}\times \mathrm{recall}}{\mathrm{precision}+\mathrm{recall}},$$

where TP, short for true positive, denotes the number of true detections; FP, short for false positive, denotes the number of false detections; FN, short for false negative, denotes the number of missed detections. The recall in Equation (2) indicates the model’s ability to detect the true HSs over all the HSs, and the precision in Equation (3) reflects the model’s ability to detect the true HSs over the whole detection. The recall and precision have a tradeoff relationship, with a higher recall implying fewer missed HS detections and a high precision implying a more confident detection of HSs. The F1-score in Equation (4) is a weighted average of the recall and precision.

### 4.2. Training Strategy

**Figure 6:**
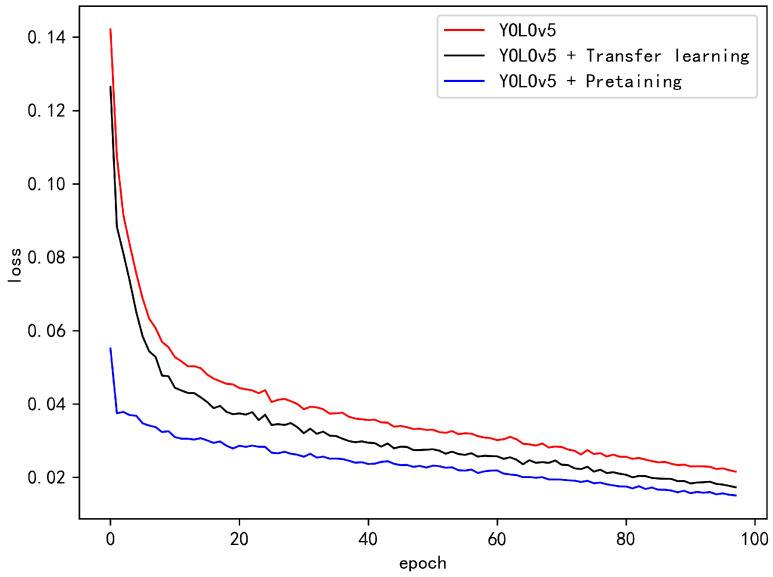
Training curve improved by transfer learning and pre-training.

Deep neural networks have suffered from heavy training times. Hence, the training parameters were set accordingly for transfer learning and pre-training: the epoch was set to 100, batch size was set to 30, image size was set to 640×640, and the IoU (intersection over union) threshold was set to 0.6. Figure 6 depicts the training curve of the loss curves with three different training strategies for Benchmark5, where the red, black and blue colors represent the training curves of directly training with Benchmark5; the transfer learning, which adapts the knowledge learned from the data in the official YOLOv5 pre-trained model on the ImageNet dataset; and the fine-tuning of the pre-trained model with Benchmark1, respectively. As given in Figure 6, the strategies used for pre-training, transfer learning and direct training are ranked in descending order in terms of both convergence and lower cost. As a consequence, with the justification in Figure 6, we employ a progressive strategy where transfer learning from the official YOLOv5 pre-trained model is adapted to Benchmark1, and the knowledge of the pre-trained Benchmark1 is adapted to Benchmarks 2–5. We also reset the epoch number to 50, which is facilitated by the proposed training strategies.

### 4.3. Result

We apply the performance indicators in Section 4.1 to evaluate the inference of the testing data in Table 2 with the training strategies, which are described in Section 4.2. To justify the effectiveness of including the SA attention mechanism in the lithography HS detection task, we compare the performance of the YOLOv5 and YOLOv5 models with and without SA. In the test phase, the IOU threshold is set to 0.8 and the confidence threshold is set to 0.6, with the caveat that a too-low confidence threshold may lead to decreased precision, while a too-high one may lead to a lower recall—hence the tradeoff between the two indicators. Additionally, there is no preference of high HS probabilities over low ones in terms of the indicators’ calculations wherever a bigger-than-the-confidence-threshold probability is detected in a prediction box. As presented in Table 3, we conducted a comparison among five object detection algorithms. In terms of recall, the Faster R-CNN demonstrated a significantly lower value of 0.7035 compared to YOLO-series algorithms. Regarding the computational efficiency, the Faster R-CNN required the longest processing time at 13.93 h, while YOLOv3, YOLOv5, and YOLOv7 exhibited identical recall rates. YOLOv5 marginally outperformed YOLOv3 in precision and F1-score metrics, whereas YOLOv7 surpassed both YOLOv3 and YOLOv5 in these two performance indicators. However, both YOLOv3 and YOLOv7 demonstrated longer computation durations than YOLOv5. Although YOLOv8 achieved an optimal computational efficiency, its recall rate of 0.9292 was deemed insufficient, considering that the primary objective of HS detection necessitates accurate identification. Through a comprehensive evaluation of all performance metrics, this research ultimately selected the YOLOv5 algorithm as the optimal solution for high-precision lithographic detection tasks.

From the indicators given in Table 4, the precisions of YOLOv5 without SA in Benchmark1 and Benchmark5, 0.9262 and 1, respectively, are marginally better than those of the proposed network with SA, 0.8933 and 0.8034, by 0.0329 and 0.1966, respectively. For Benchmarks 2–4 and 6, the proposed Yolov5 model with SA improves the precisions of the YOLOv5 model without SA from 0.9188, 0.4386, 0.1817 and 0.35 to 0.9689, 0.6627, 0.8028 and 0.8277, respectively, with large margins of 0.2214, 0.6211 and 0.4777 in Benchmarks 3, 4 and 6. Similar observations with the F1-score are also demonstrated in Table 4, where the network with SA outperforms the one without in Benchmarks 3, 4 and 6, but it is slightly outperformed in Benchmarks 1 and 5. Therefore, the inclusion of SA improves the overall detection ability of lithography HSs.

**Figure 7:**
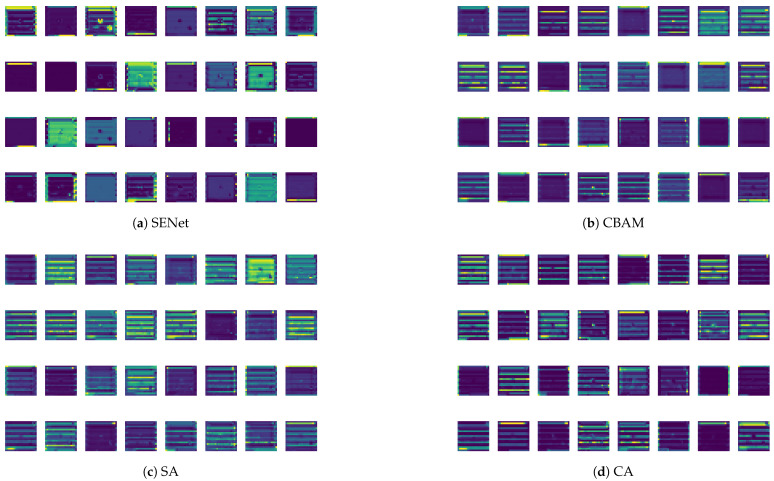
Extraction of different attention mechanisms’ feature maps from C1 in Figure 4.

We further explore the performance of including different attention mechanisms in the YOLOv5 model for the lithography HS detection task, where the SA boxed with light purple in the CBS-SA block module in Figure 4 is replaced by SENet [30], CBAM [25] and CA (channel attention). Both being channel-based, SENet applies global average pooling for squeezing while CA applies additional global max pooling for improved higher-lever feature extraction. CBAM is a hybrid attention mechanism, including CA and SA. The training and testing environments of all the models embedding different attention mechanisms are kept consistent for the cogency of the comparison. From the results given in Table 5, the precisions with all the mechanisms for Benchmarks 1 and 2 are similar with a maximum difference of less than 0.04. For Benchmark5, the models with CBAM and CA top the precision ranking jointly with a score of 1, outscoring 0.8034 with SA and 0.7593 with SENet. For Benchmarks 3, 4 and 6, the precisions with SA, 0.6627, 0.8028 and 0.8277, outperform those of other mechanisms by large margins of 0.0832, 0.0856, and 0.4375 over SENet; 0.2254, 0.2437, and 0.4565 over CBAM; and 0.2806, 0.2252, and 0.5945 over CA, respectively. As shown in Figure 7, we extracted the C1 feature map from Figure 4. Given that the lithography HS region in the mask is located at the center, we found that SA can significantly focus on the HS region and accurately extract the detailed features of this region through a comparison of the feature maps generated by four different attention mechanisms. Meanwhile, the CBAM also shows a certain degree of attention with regard to the HS region, but it is slightly inferior to SA in terms of detailed feature extractions. In contrast, although the SENet and the CA demonstrate the ability to perceive entire images globally, they perform poorly in terms of attention with regard to the HS region and capturing detailed features. In the lithography process, sensitive layout patterns exhibit prominent spatial characteristics. However, there is no direct correspondence between the channel dimension of the feature maps and the physical properties of lithography. For tasks like HS detection, which highly rely on spatial information, SA directly constructs a geometric sensitivity model to avoid the potential global statistical biases introduced by the channel attention mechanism. We believe including SA within the YOLOv5 model enhances the learning ability of HS and NHS geometric features.

Additionally, we discuss the results of all test cases, comparing the proposed YOLOv5+SA model with the contemporary HS detection methods in the literature. Table 6 presents the detailed detection performance. Wang et al. [31] developed the high-resolution network HR-Net18 with pre-training for HS detection. Shin et al. [14] integrated the powerful classification performance of convolutional neural networks (CNNs), data augmentation during training, candidate region selection, and density-based scan (DBSCAN) clustering for HS detection tasks. Zhou et al. [13] studied the feasibility of deep learning in lithographic HS detection, and they used hybrid data augmentation to compensate for the lack of HS data and the large layout size. Data compression and pre-training are also included to improve the detection performance. Chen et al. [32] integrated the Squeeze-and-Excitation (SE) attention mechanism with the Efficient Channel Attention (ECA) mechanism to enhance the efficiency and performance of deep learning models in extracting hotspot features.

It is noted that although excelling in precision, Wang’s method obtains a significantly lower recall number than Shin’s, Zhou’s and our method due to low hit ratios. Our approach with YOLOv5+SA presents the best average results among all the approaches. Our approach exhibits perfect hit ratio in terms of recall and significantly improves precision and the F1-score of Shin’s and Zhou’s approaches by large margins for all benchmarks. For instance, with Benchmark6, the precision and F1-score gains are (0.6057, 0.3527) and (0.5507, 0.2707) from Shin’s and Zhou’s approaches, respectively. Aside from perfect recall, our approach has a more stable precision and F1-score in the ranges of 0.6627 to 0.9689 and 0.7972 to 0.9842. Although Chen’s method outperforms our method in terms of the F1-score and precision, our primary goal is to identify hotspots for the lithography hotspot detection task. Our method counts all the networks with a confidence level higher than 0.6 as detected hotspots, without comparing the probabilities of NHSs. Consequently, our F1-score and precision are lower than those of Chen’s method.

### 4.4. HS Detection Test

**Figure 8:**
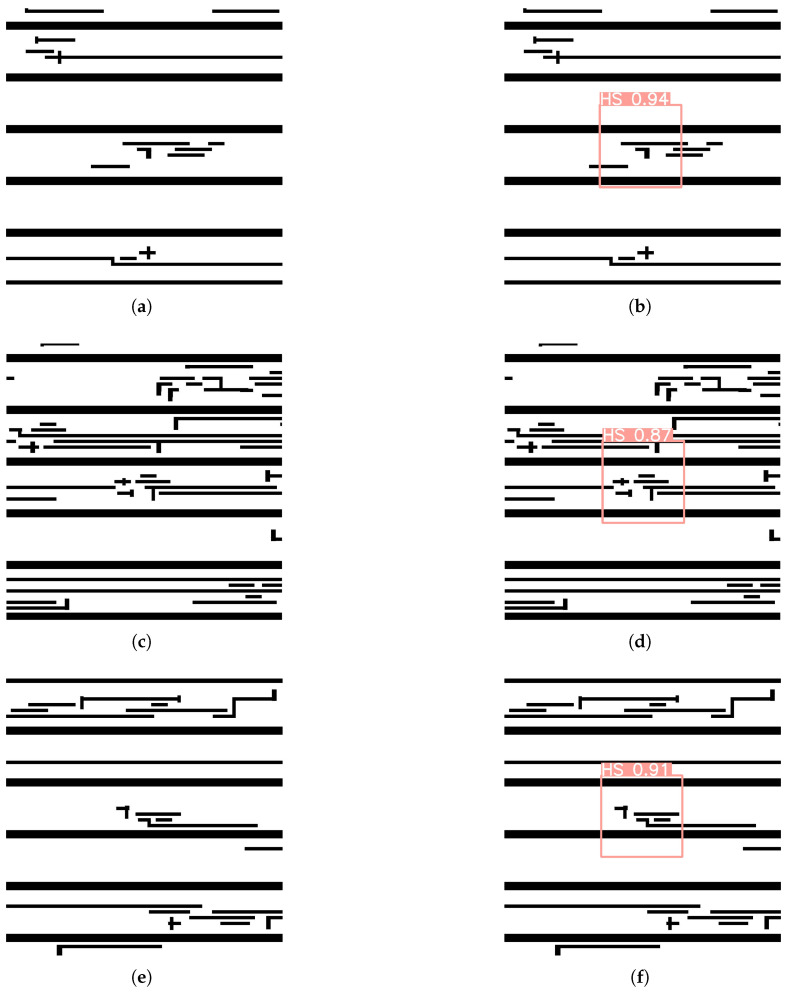
Results of lithography HS detection using trained YOLOv5+SA. (**a**) First original mask. (**b**) Detection result image of the first original mask. (**c**) Second original mask. (**d**) Detection result image of the second original mask. (**e**) Third original mask. (**f**) Detection result image of the third original mask.

Figure 8a,c,e show the original masks. When the layout is input into the trained network model, the output result of the model is as shown in Figure 8b,d,f. By observing the detection result image, we can clearly see the that the model has accurately marked the HS area in the original mask as a rectangular box, and the probability of a HS within this boxed area is shown in the detection result image. This demonstrates that YOLOv5+SA is highly effective in detecting HS regions within the original mask.

## 5. Conclusions

We develop an improved YOLOv5 framework for lithography HS detection, incorporating a spatial attention mechanism in the Backbone module to improve the network’s learning ability of HS geometric features. Layout flipping tailored for lithographic image formations is designed to address HS and NHS imbalances. Meanwhile, the proposed YOLOv5+SA network is trained and validated with the dataset of the ICCAD 2012 contest benchmarks, where a progressive strategy with transfer learning and pre-training is applied for data insufficiency and accelerated convergence. The experimental results support the inclusion of the SA mechanism, showing improved performance in HS detection, with an average recall rate, precision and F1-score of 1.0, 0.8277 and 0.9057, respectively. Compared with contemporary HS detection methods, our method showcased superior and stable HS detection in all the benchmarks, highlighting the effectiveness and feasibility of integrating attention mechanisms with YOLO structures. The proposal of our scheme greatly improves the detection efficiency of lithography hotspots, laying the foundation for optical proximity correction. Our work further demonstrates the potential of deep learning in the field of computational lithography and lays the technical groundwork for the application of deep learning in computational lithography.

## Figures and Tables

**Table 1:** Benchmark statistics.

Name	Technology (nm)	Training Dataset		Test Dataset	
		HS	NHS	HS	Area (nm^2^)
Benchmark1	32	99	340	226	12,516
Benchmark2	28	174	5285	498	106,954
Benchmark3	28	905	4642	1796	122,565
Benchmark4	28	95	4453	177	82,010
Benchmark5	28	26	2716	41	49,583
Benchmark6	28	1200	17,096	2512	361,112

**Table 2:** Benchmark statistics with data augmentation.

Name	Training Dataset		Validation Dataset		Test Dataset	
	HS	NHS	HS	NHS	HS	Area (nm^2^)
Benchmark1	743	2579	32	142	226	12,516
Benchmark2	1251	4776	141	509	498	10,694
Benchmark3	800	4187	109	455	1808	122,565
Benchmark4	650	4013	78	439	177	82,010
Benchmark5	187	2445	21	271	41	49,583
Benchmark6	8558	15,378	941	1718	2524	361,112

**Table 3:** Comparison of different methods.

Name	Methods	Recall	Precision	F1-Score	Runtime (h)
Benchmark1	Faster R-CNN	0.7035	0.1513	0.2490	13.93
	YOLOv3	1	0.9150	0.9556	6.058
	YOLOv5	1	0.9262	0.9617	2.579
	YOLOv7	1	0.9658	0.9827	6.608
	YOLOv8	0.9292	0.9333	0.9313	1.239

**Table 4:** Results with and without SA.

Name	Methods	Recall	Precision	F1-Score
Benchmark1	YOLOv5	1	0.9262	0.9617
	YOLOv5+SA	1	0.8933	0.9436
Benchmark2	YOLOv5	1	0.9188	0.9577
	YOLOv5+SA	1	0.9689	0.9842
Benchmark3	YOLOv5	1	0.4386	0.6097
	YOLOv5+SA	1	0.6627	0.7972
Benchmark4	YOLOv5	1	0.1817	0.3076
	YOLOv5+SA	1	0.8028	0.8906
Benchmark5	YOLOv5	1	1	1
	YOLOv5+SA	1	0.8034	0.8913
Benchmark6	YOLOv5	1	0.35	0.5184
	YOLOv5+SA	1	0.8277	0.9057

**Table 5:** Comparison of different attention mechanisms.

Name	Methods	Recall	Precision	F1-Score
Benchmark1	SENet	1	0.9262	0.9617
	CBAM	1	0.9187	0.9576
	SA	1	0.8933	0.9436
	CA	1	0.9187	0.9576
Benchmark2	SENet	1	0.9670	0.9832
	CBAM	1	0.9708	0.9852
	SA	1	0.9689	0.9842
	CA	1	0.9468	0.9727
Benchmark3	SENet	1	0.5795	0.7338
	CBAM	1	0.4373	0.6085
	SA	1	0.6627	0.7972
	CA	1	0.3821	0.5530
Benchmark4	SENet	1	0.7172	0.8353
	CBAM	1	0.5591	0.7172
	SA	1	0.8028	0.8906
	CA	1	0.5776	0.7322
Benchmark5	SENet	1	0.7593	0.8632
	CBAM	1	1	1
	SA	1	0.8034	0.8913
	CA	1	1	1
Benchmark6	SENet	1	0.3902	0.5614
	CBAM	1	0.3712	0.5414
	SA	1	0.8277	0.9057
	CA	1	0.2332	0.3781

**Table 6:** Detailed performance comparison with contemporary methods.

Name	Methods	Recall	Precision	F1-Score
Benchmark1	Wang	0.631	0.995	0.771
	Shin	0.951	0.358	0.520
	Zhou	0.995	0.324	0.489
	Chen	0.971	0.976	0.974
	Ours	1	0.8933	0.9436
Benchmark2	Wang	0.908	0.921	0.914
	Shin	0.988	0.216	0.354
	Zhou	0.986	0.702	0.82
	Chen	0.993	0.893	0.941
	Ours	1	0.9689	0.9842
Benchmark3	Wang	0.897	0.980	0.937
	Shin	0.975	0.199	0.331
	Zhou	0.982	0.443	0.64
	Chen	0.953	0.861	0.905
	Ours	1	0.6627	0.7972
Benchmark4	Wang	0.859	0.886	0.873
	Shin	0.938	0.157	0.269
	Zhou	0.972	0.355	0.52
	Chen	0.997	0.927	0.960
	Ours	1	0.8028	0.8906
Benchmark5	Wang	0.651	0.948	0.771
	Shin	0.927	0.181	0.303
	Zhou	0.98	0.549	0.704
	Chen	0.999	0.956	0.978
	Ours	1	0.8034	0.8913
Benchmark6	Wang	-	-	-
	Shin	0.955	0.222	0.355
	Zhou	0.983	0.475	0.635
	Chen	-	-	-
	Ours	1	0.8277	0.9057

## Data Availability

The original contributions presented in the study are included in the article, further inquiries can be directed to the corresponding author.
